# Causation and Treatment of Empyema, Renvers

**Published:** 1890-04

**Authors:** 


### RENVERS.—CAUSATION AND TREATMENT OF EMPYEMA.

Charité Annalen, 1890, XIV., pp. 108-227. Resumé, by Lühe, in *Centralblatt für Chirurgie.*

TRANSLATED BY M. B. HUTCHINS, M. D.

By means of a number of clinical histories, R. endeavors to sift the reasons for varied opinions of methods of operation for the removal of pus in empyema. First in order, categorically, will be single cases. There are to distinguish:

(1) Metapneumonic empyema with pneumonia-cocci, alone, in the pus. Here, at first, results could be expected from simple puncture and aspiration, with the addition of drainage if necessary.

(2) Metapneumonic empyemæ with presence of staphylococci and streptococci, in addition to the diplococci of pneumonia. The lungs will be fixed upon as the entrance-portal for the specific pus-cocci also. Here a radical operation with rib-resection could scarcely be avoided.

(3) Empyema which particularly develop putrid bronchitis, in addition to ulcerative processes in the lungs. Radical operation unavoidable. In one of the cases mentioned the fistulous opening in the lung had to be enlarged through incision of the thickened lung tissue, in order to make, in the lung itself, a passage continuous with the opening into the empyemic cavity. With this there occurred considerable bleeding, which only ceased after tightly packing with iodoform gauze, which, however, was evident for several days in the discharge of bloody detritus. The use of the thermo-cautery might obviate this bleeding.

(4) Septic, secondary, empyema, mostly puerperal, offer only bad prospects for the radical operation. Comparatively, the unilateral, subphrenic suppurations offer the best prognosis when it is possible to empty the primary abscess into the abdominal cavity.

(5) Suppurations occurring in tubercular cases naturally divide themselves into those which arise accidentally without being of tubercular nature, and those directly dependent upon that process. In the first, the prospects of the radical operation would only be dimmed by the bad general condition, but here, even, puncture and drainage may bring about healing. On the other hand, in most cases, even with the radical operation, for tubercular disease of the pleura, as well as tubercular pneumothorax, one must be content with a relative healing and fistula-formation. Then the deficient expansion of the lung will sometimes necessitate Schede's thoraco-plastic operation. Altogether, the later statistics, especially those of Küster, demonstrate that one is not justified in declaring primarily every empyema of a tubercular subject a *noli me tangere*.

---

WHOOPING-COUGH.—A two per cent. watery solution of resorcin sprayed into the nose, throat, and larynx every two hours is a remarkably efficient agent in the treatment of whooping-cough, according to Dr. J. W. Farlow, of Boston.

---

PROF. EDWARD VON WAHL, of Dorpat, one of the editors of the *St. Petersburger medicinische Wochenschrift*, died on January 29, in consequence of an accident he met with a short time ago, by which several of his ribs were fractured.

---

AN EXCELLENT OINTMENT FOR ACNE—May be formed by taking two parts of resorcin and boracic acid, and adding to it twenty parts of vaseline. This should be rubbed in night and morning, after plenty of hot soap and water and a good rough towel has been used.

